# Unmitigated Surgical Castration in Calves of Different Ages: Electroencephalographic and Neurohormonal Findings

**DOI:** 10.3390/ani11061791

**Published:** 2021-06-15

**Authors:** Luciana Bergamasco, Lily N. Edwards-Callaway, Nora M. Bello, Sage Mijares, Charley A. Cull, Ruby A. Mosher, Johann F. Coetzee

**Affiliations:** 1 Department of Clinical Sciences, College of Veterinary Medicine, Kansas State University, Manhattan, KS 66506, USA; 2 Department of Animal Science and Industry, College of Agriculture, Kansas State University, Manhattan, KS 66506, USA; lily.edwards-callaway@colostate.edu; 3 Department of Statistics, College of Art and Sciences, Kansas State University, Manhattan, KS 66506, USA; nbello@ksu.edu; 4 Department of Animal Sciences, College of Agricultural Sciences, Colorado State University, Fort Collins, CO 80523, USA; mijaress@rams.colostate.edu; 5 Department of Diagnostic Medicine/Pathobiology, College of Veterinary Medicine, Kansas State University, Manhattan, KS 66506, USA; charley@mvsinc.net (C.A.C.); rubymosher60@gmail.com (R.A.M.); 6 Department of Anatomy and Physiology, College of Veterinary Medicine, Kansas State University, Manhattan, KS 66506, USA; jcoetzee@vet.k-state.edu

**Keywords:** age, calves, electroencephalography, pain, substance P, surgical castration

## Abstract

**Simple Summary:**

Castration is a painful procedure that is commonly performed on cattle without analgesia. Although castration is considered to be more painful in older calves, studies examining the effect of age on electrophysiological and neurohormonal responses to pain under the same experimental conditions are limited. There are several limitations to providing pain mitigation for castration, one being the lack of available approved analgesics for use in alleviating the pain associated with castration in the United States. It is necessary to validate methods of pain assessment in cattle to support the development of pain relief drugs. The aim of this study was to investigate the effect of unmitigated surgical castration on the electroencephalography (EEG) responses and plasma substance P (SP) concentrations in calves, without pain relief across different age groups. Age, time, and procedure (castration or a simulated castration) impacted outcomes. The findings suggest that surgical castration without pain relief causes variations in EEG responses and in SP concentrations and that these responses are age related.

**Abstract:**

Castration is a common management procedure employed in North American cattle production and is known to cause a pain response. The present study was designed to investigate the effect of unmitigated surgical castration on the electroencephalography (EEG) responses and plasma substance P (SP) concentrations in calves of different ages under the same experimental conditions. Thirty male Holstein calves in three age categories [<6 weeks (6W); 3 months (3M); 6 months (6M); 10 calves per age group] were used in the study. Calves were subjected to a simulated castration session (SHAM) followed 24 h later by surgical castration (CAST) without analgesia. An EEG analysis was performed before the procedure (i.e., baseline), at treatment, and 0–5, 5–10, and 10–20 min post-treatment for both SHAM and CAST, respectively. Blood samples were collected immediately prior to both treatments (time 0) and again at 1, 2, 4, 8, and 12 h after both treatments. The EEG results showed a three-way interaction between treatment, age, and time for delta and beta absolute power, beta relative power, total power, and median frequency (*p* = 0.004, *p* = 0.04, *p* = 0.04, *p* = 0.03, and *p* = 0.008, respectively). Following CAST, EEG total power decreased, and median frequency increased relative to SHAM in 6W and 3M calves only following treatment. For 6W and 3M calves, delta and beta absolute power increased at CAST and at later time points relative to SHAM. Marginal evidence for two-way interactions was noted between time and treatment and between age and treatment on the concentration of SP (*p* = 0.068 and *p* = 0.066, respectively). Substance P concentrations decreased in CAST treatment compared to SHAM at the later times (8 h: *p* = 0.007; 12 h: *p* = 0.048); 6W calves showed lower SP concentration at CAST relative to SHAM (*p* = 0.017). These findings indicate variation in EEG responses and in SP concentrations following unmitigated surgical castration in calves and that these responses may be age specific. These EEG findings have implications for supporting the perception of the pain associated with surgical castration in young calves and emphasize the urgency of pain mitigation strategies during routine husbandry practices such as castration, as typically implemented in North American cattle management.

## 1. Introduction

Castration of calves is a common management practice used by cattle producers in the United States. Given that surgical castration causes a pain response, strategies for minimizing pain and stress are advocated [1,2,3]. In the United States, pain control for castration is voluntary. Currently in the United States there is only one drug approved by the FDA, flunixin meglumine, for the control of pain in cattle specifically related to interdigital phlegmon (e.g., foot rot). Therefore, use of analgesics for alleviating pain associated with castration in cattle constitutes extra-label drug use and must be used under the guidance of a veterinarian. Analgesic agents are typically administered before or at the time of a painful procedure, such as castration or dehorning, to relieve the pain and distress associated with the event. Substances available to administer to cattle to alleviate the pain associated with painful procedures include local anesthetics, nonsteroidal anti-inflammatory drugs (NSAIDs), opioids, alpha2-agonists, and N-methyl-D-aspartate (NMDA) receptor agonists [4].

In a survey study published in 2010, 21% of bovine veterinarians reported administering analgesia at the time of castration [5]. A recent survey conducted to evaluate how pain management has changed in the cattle industry over the past decade indicated that 32.5% to 47.4% of veterinarians use pain mitigation most of the time or always during surgical castration, varying by calf age [6]. The percentage of producer respondents that utilize local analgesia most of the time or always during surgical castration was considerably lower than veterinarian respondents; producers utilized pain mitigation during surgical castration for calves 2 to 12 months of age 13.1% of the time, while veterinarians reported using pain mitigation 38.8% of the time [6]. Low analgesic use in farm animals has been partially attributed to a lack of methods for identifying and measuring pain in farm animals [4,7] in addition to cost, convenience of treatment, and regulatory limitations [6,8]. There are several physiological measurements that have been shown to be effective in measuring pain response in humans and animals. Electroencephalography (EEG) measurements are used to measure pain in human pain perception studies [9,10,11,12]. The power of different EEG bandwidths is associated with a pain response [9]. Similar EEG changes have been reported in animals in response to pain [13,14,15]. Activity is measured by four bands, namely low-frequency δ (delta) and θ (theta) and high-frequency α (alpha) and β (beta). In general terms, a typical EEG response to pain involves a decrease in total spectral power (Ptot), an increase in 95% spectral edge (the 95th percentile of the power spectrum), and an increase in median frequency (the frequency that divides the entire EEG spectrum in two parts of equal absolute power [15]). This response is attributed to a decrease in low-frequency activity and an increase in high-frequency activity. This phenomenon is termed desynchronization and has been associated with increased arousal [15]. A study performed in cattle during castration showed an association between EEG responses and cortisol concentrations, thereby suggesting that EEG may be useful in assessing increases in brain electrical activity associated with pain [13]. EEG has also been used to assess brain activity in calves using different slaughter techniques. EEG recordings in captive bolt stunned animals displayed high-amplitude, slow-frequency waves after stunning, indicating immediate unconsciousness [16], similarly found by Lambooy and Spanjaard [17] and Zulkifli et al. [18].

Furthermore, substance P (SP) is a neuropeptide produced in a subset of peripheral neuron cell bodies localized in dorsal roots and plays a pivotal role in the transmission of noxious stimuli in the spinal cord [19]. Increases in plasma SP concentration post-castration have been reported in cattle [20,21,22,23], suggesting that SP levels might be associated with pain.

The present study was designed to assess EEG and SP concentrations in calves of different ages subjected to unmitigated castration in order to support pain control for routine husbandry practices. We hypothesize that EEG and SP are physiological pain-related indicators in cattle that are affected by unmitigated castration in an age-specific manner, i.e., a reduced EEG response and lower SP in younger animals. The aim of this study was (1) to compare EEG and SP concentrations in calves undergoing surgical castration relative to a simulated castration session and (2) to assess differences in EEG and SP concentrations between age groups of calves during simulated and surgical castrations.

## 2. Materials and Methods

This protocol was approved by the Institutional Animal Care and Use Committee at Kansas State University (Protocol #2831) and was conducted during the Summer of 2010. This study was a component of a federally funded grant (USDA-CSREES NRI Award No. 2009-65120-05729) exploring differences in pain response in varying ages of cattle; a portion of the results are reported in this paper. The United States cattle industry guidelines suggest castrating calves at the youngest age possible [1,2,3], indicating that prior to 3 months of age is ideal [1]. Based on these recommendations, we selected three industry-relevant age groups to use in this study.

### 2.1. Animals and Housing

Male Holstein calves of 6 weeks (6W; 52 ± 9 kg), 3 months (3M; 89 ± 5 kg), and 6 months (6M; 139 ± 11 kg) of age (10 calves per age group) were enrolled in the study and housed at the Kansas State University Beef Cattle Research Center (BCRC; Manhattan, KS, USA), where calves were subjected to a simulated castration (SHAM) followed by a surgical castration (CAST) 24 h later, subsequently described. Calves were obtained from one Kansas dairy herd and acclimated for 10 days prior to study initiation. Upon arrival at the BCRC, study animals received a four-way modified-live viral respiratory disease vaccine (Bovishield Gold, Pfizer, New York, NY, USA) and were administered oxytetracycline (Noromycin 300 LA, Norbrook Laboratories Station Works, Newry, Co. Down, N. Ireland; 9 mg/kg body weight IM).

The 3M and 6M treatment groups were housed by age group (e.g., treatment group) on outdoor concrete pads (9.8 m × 18.3 m) with a partial roof over straw bedding. Throughout the acclimation and study period, calves were maintained on water and grass hay ad libitum with a grain-based supplement provided at 3–4 kg/head/day. Unweaned < 6W calves were housed nearby in individual contiguous wire-panel enclosures (1.6 m × 5.3 m) and were fed a milk replacer (Maxicare, Land O’Lakes, Animal Milk Products Co. 039, Shoreview, MN 55126-2910, USA) with ad libitum water and starter ration (Herd Maker Supreme B90, Land O’Lakes, Animal Milk Products Co. 039, Shoreview, MN 55126-2910, USA). During acclimation, the calves were individually restrained for approximately 30 min daily with a rope halter and head gate to accustom them to subsequent sampling procedures.

Within each age group, animals were blocked by body weight and scrotal circumferences and were randomly assigned to a processing date (e.g., the day the experimental procedures started) and an order of processing within the day to avoid confounding effects. After the acclimation period, calves were submitted to a simulated castration session (SHAM) on their assigned processing date followed 24 h later by surgical castration session (CAST). These sessions were intended to allow for differentiation of the castration-inflicted pain from an overall distress response associated with handling and management.

### 2.2. Jugular Catheterization

Calves were individually restrained in head gates and fitted with rope halters approximately 12 h prior to study commencement. Following restraint, the area over the jugular vein was clipped and disinfected using 70% isopropyl alcohol and povidone iodine swabs. The catheter site was infiltrated with 2% lidocaine injection [Lidocaine Hydrochloride Injection, USP (2%) (20 mg/mL), Hospira Inc., Lake Forest, IL, USA] prior to performing a small skin incision to facilitate placement of a 14 G × 130 mm extended use catheter (MILACATH^®^, MILA International, Florence, KY, USA), which was sutured to the skin using #3 nylon suture (Braunamid^®^, Braun, Bethlehem, PA, USA) for the duration of the study. Catheter patency was maintained using a heparin saline flush containing 3 USP units heparin sodium/mL saline (Heparin Sodium Injection, Baxter Healthcare, Deerfield, IL, USA).

### 2.3. Experimental Procedure

Experimental procedures were conducted between 06:00 and 10:30 a.m., at 45 min intervals. No pain medication was used, and all surgical procedures were performed by the same experienced operator. Calves were restrained in a chute with head movement limited by a halter drawn close to a table attachment (For-Most Livestock Equipment, Hawarden, IA, USA). The location where experimental procedures occurred was not visible to study animals from their home pens. During simulated castration (SHAM) and surgical castration (CAST), the scrotum was washed with chlorhexidine disinfectant. In SHAM, the testes within the scrotum were firmly grasped, and ventral traction was applied for approximately 20 s. This time point is indicated as “treatment” in the study timeline. In CAST, surgical castration was performed by quickly removing the lower one-third of the scrotum with a sharp, disinfected scalpel. The testes and spermatic cords were exteriorized by blunt dissection followed by manual traction until the spermatic cord and connective tissue ruptured. This time point is indicated as “treatment” in the study timeline. Calves were continuously monitored for pain for 8 h following surgery, and then twice daily for 7 days. Signs of excessive pain were based on the evaluation of attitude, gait, posture, appetite, lying and scrotal swelling. Although rescue analgesia (flunixin meglumine, 2.2 mg/kg IV BID; Banamine Injectable Solution, 50 mg/mL, Merck Animal Health, Summit, NJ 07901, USA) was available for calves exhibiting signs of excessive pain, none required treatment.

### 2.4. EEG Recordings

Following the acclimation period, calves were restrained in a chute with a head table attachment. Electrodes were placed transcutaneously in a 12-channel montage (F3, F4, T3, C3, Cz, C4, T4, P3, Pz, P4, O1, O2; odd number = left hemisphere; even number = right hemisphere [13]) to record EEG (Sandman Spyder, Tyco Healthcare, Puritan Bennet Ltd., Kanata, ON, Canada) following the methodology of Bergamasco et al. [13]. To ensure good electrical contact with the electrodes, the scalp was defatted by rubbing vigorously with ethyl alcohol. No local infiltration of lidocaine was performed around electrode placement sites. Electrode placement was adapted from suggestions by Takeuchi et al. [24], and electrodes were placed over the frontal, central, temporal, parietal, and occipital regions as well as along the median sagittal line. Referential and bipolar montages (F3-T3, T3-O1, F3-C3, C3-P3, P3-O1, F4-C4, T4-O2, F4-C4, C4-P4, P4-O2, T3-C3, C3-Cz, Cz-C4, C4-T4) were used. The acquisition parameters to record bio-electrical activity were set as follows: sensitivity = 5 µV/mm; time constant = 0.3 s; high filter (Hf) = 70 Hz; notch filter inserted; reference = on the bridge of the nose; ground = caudally to the external occipital protuberance; electrode impedance < 3 kΩ; sampling rate = 256 Hz. Fourteen EEG needles (12 mm × 28ga, Chalgren Enterprise, Inc., Gilroy, CA, USA) were used as active, reference, and ground electrodes. Electrocardiogram (EKG) and respiratory rates were recorded via polygraphic electrodes (EKG: sensitivity = 70 µV/mm, time constant = 0.1 s, Hf = 30 Hz; respiration: sensitivity = 20 µV/mm, time constant = 0.3 s, Hf = 30 Hz) connected to alligator clips (thin cable for bridge electrode, Bionen S.a.S., Italy) and to a respiratory effort system (Sleepmate, MVAP, Newbury Park, CA, USA).

EEG recording started when electrode placement was completed, about 10 min after calves were restrained. The total recording time was 30 min, including calibration and the initial impedance check. The time periods of EEG recording included baseline (10 min before treatment; Base), scrotum manipulation (Scrot), treatment (actual cutting and pulling; Trt), immediate recovery (0–5 min post-treatment; R05), middle recovery (5–10 min post-treatment; R510), and late recovery (10–20 min post-treatment; R1020). A 10 min baseline period was selected as it would provide enough 2 s artifact-free epochs, which is necessary to summarize the most relevant information about a particular EEG sample in terms of quantitative measures derived from power spectrum analysis. All time designations in SHAM and CAST were aligned to Trt, and the EEG recorded throughout the experimental procedure (e.g., from Base to R1020 continuously). As the EEG time periods started and stopped during the recordings, specific marks (e.g., Base) were inserted in the ongoing EEG recordings. Additionally, the castration timeline included specific marks related to the beginning of Scrot and Trt. An example of EEG output with these specific marks can be found in Coetzee [8]. The EEG data were stored in the acquisition station for later analysis.

### 2.5. EEG Analysis

Electroencephalography visual and quantitative analyses were conducted by an experienced operator blinded to treatment and age classification. Before performing the quantitative analysis, all EEGs were examined visually to evaluate the background activity and artifacts. Special emphasis was given to artifact detection and elimination because they strongly affect the frequency analysis of the EEG [25,26]. Ocular movements, cardiovascular and muscular activity, physiological rhythmic movements, or recording environmental artifacts were noted and manually rejected. Data from two of the 3M calves at SHAM were excluded from any further analysis due to unacceptable levels of artifact contamination of the EEG.

Bio-electrical activity was analyzed using an integrated software program (Software Spiderline, Version 2.32, EBNeuro, Firenze, Italy). For all calves, a minimum of 30 measurements of 2-s artifact-free epochs were visually selected for each designated time period of the EEG timeline. Fast Fourier Transform was calculated for each channel. The spectral bands of delta (0.5–4.0 Hz), theta (4.1–8.0 Hz), alpha (8.1–12.0 Hz), and beta (12.1–30.0 Hz) were calculated and expressed as absolute power (AP; µV2) and relative power (RP; %) for the four frequency bands. The total power (Ptot; µV2) and median frequency (F50; Hz) of the entire spectrum (0.5–30.0 Hz) were also calculated. The AP corresponds to the area under the curve of the spectrum between the two frequencies that define the bandwidth. The Ptot is the area under the entire spectral curve. The RP is defined as a ratio between a given band’s absolute power and the Ptot, multiplied 100 times [27].

### 2.6. Substance P

Blood samples for SP analysis were collected using the above-described catheters immediately prior to treatment (time 0) and again 1, 2, 4, 8, and 12 h after treatment for both SHAM and CAST. Blood was collected into EDTA K3 tubes (Vacuette 6 mL K3E Tubes, Greiner Bio-One, Kremsmünster, Austria) for SP analysis. A 20 mM solution of benzamidine was prepared in water and 300 µL was added to each EDTA K3 tube for a final concentration of 1 mM benzamidine in whole blood to act as a protease inhibitor. These tubes were stored on ice for no more than 30 min before being centrifuged at 1500× *g* for 10 min. The plasma was pipetted off with 3 mL transfer pipettes and stored in 2 mL cryogenic vials and stored at −80 °C until analysis. Samples were analyzed for SP concentrations using a validated analytical method and in the same laboratory as previously described [20]. The laboratory technician performing the analysis was blinded to treatment and age classification.

### 2.7. Statistical Analysis

A general linear mixed model was fitted to each response variable included in the study.

Some of the responses were log-transformed to ensure variance stabilization, namely EEG single frequency (theta, alpha, and beta bands) absolute and relative power, EEG total power, and substance P concentrations. Delta band RP was subjected to a quadratic transformation for similar reasons.

In all models, the linear predictor included the fixed effects of treatments (namely the experimental procedures: SHAM vs. CAST), age, time points, and all two- and three-way interactions. The random effect of calf nested within age group was included in the model to recognize calf as the experimental unit for age, and the blocking factor for treatment. Moreover, the random effect of calf-by-treatment combination recognized calf as the experimental unit for treatment, and the random effect of calf-by-treatment-by-time combination accounted for repeated measures over time, while the residual term recognized technical replication, where applicable. For EEG analyses, spatial variance-covariance structures were fitted at the residual level to account for the spatial montage of channels used to record EEG. In all cases, residual model assumptions were thoroughly checked and were considered to be reasonably met. The EEG model was fitted using the MIXED procedure of SAS (Version 9.2, SAS Institute Cary, NC, USA). The SP models were fitted using the GLIMMIX procedure of SAS (Version 9.2, SAS Institute, Cary, NC, USA), implemented using Newton-Raphson with ridging as the optimization technique. Results are presented in the original data scale (estimated least-square means and corresponding standard errors or 95% confidence intervals). For those response variables that were analyzed in a transformed (e.g., log) scale, point estimates and confidence intervals are reported in the original scale following back-transformation. Relevant pairwise comparisons were conducted using Tukey-Kramer or Bonferroni adjustments, depending on the level of inference, to avoid inflation of Type I error rate due to multiple comparisons. A significance level of α = 0.05 was used to determine statistical differences, as well as α ≤ 0.10 for assessing effects with marginally significant differences.

## 3. Results

### 3.1. Electroencephalography

**Figure 1:**
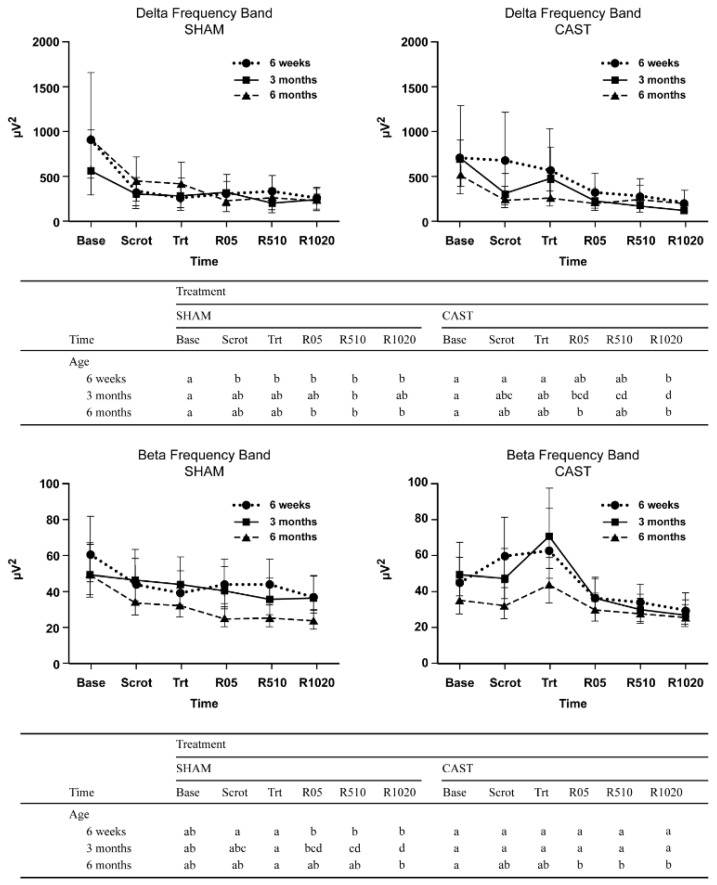
Estimated least-square means and 95% confidence intervals of absolute power (µV^2^) of delta and beta frequency bands for 6-week—(6W), 3-month—(3M), and 6-month-old—(6M) calves undergoing simulated castration (SHAM) and surgical castration (CAST) treatments. Time points evaluated consist of baseline (Base), scrotum manipulation (Scrot), treatment (Trt), immediate recovery (R05), middle recovery (R510), and late recovery (R1020). Letters (a, b, c, d) indicate a significant difference (*p* < 0.05) between the time points within each combination of age and experimental treatment along each row in the table.

A three-way interaction was noted between treatment, age, and time (*p* = 0.004; Figure 1) for AP of the delta band. For all age groups in SHAM, the estimated delta AP was greatest at baseline and decreased thereafter to a plateau. In contrast, age-specific patterns in AP were apparent in the CAST treatment. In particular, for 6W calves undergoing CAST, delta AP at treatment time (Trt) was greater relative to SHAM (*p* = 0.03). This difference was not apparent for older calves (*p* = 0.54 and 0.74 for 3M and 6M calves, respectively). Moreover, for 6W and 3M calves undergoing CAST, delta AP was greater at treatment time relative to later recovery times (*p* < 0.05).

A three-way interaction was identified between treatment, age, and time (*p* = 0.04; Figure 1) for AP of the beta band. Specifically, CAST 6W and 3M calves at the time of treatment had greater beta AP than SHAM (*p* = 0.02 and *p* = 0.05, respectively). Moreover, calves of all ages undergoing CAST had greater beta AP at treatment time relative to later recovery times (i.e., R05, R510, and R1020 for 6W and 3M calves, *p* < 0.02; R1020 for 6M calves, *p* < 0.01); these time differences were not apparent at SHAM for any age category.

**Figure 2:**
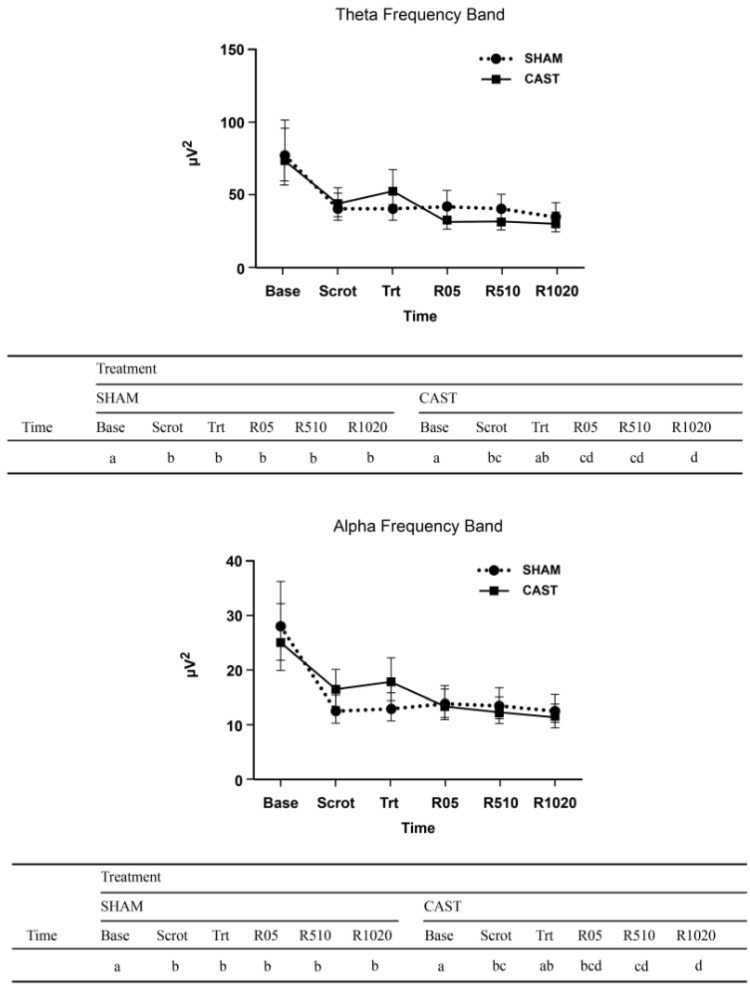
Estimated least-square means and 95% confidence intervals of absolute power (µV^2^) of theta and alpha frequency bands for 6-week—(6W), 3-month—(3M) and 6-month-old—(6M) calves undergoing simulated castration (SHAM) and surgical castration (CAST) treatments. Timepoints evaluated consist of baseline (Base), scrotum manipulation (Scrot), treatment (Trt), immediate recovery (R05), middle recovery (R510), and late recovery (R1020). The letters (a, b, c, d) indicate a significant difference (*p* < 0.05) between time points within a given treatment after accounting for age effects.

For theta and alpha AP, a two-way interaction between treatment and time was noted (*p* = 0.01 and *p* = 0.04, respectively), whereby regardless of age, the dynamics of the response over time differed between SHAM and CAST (Figure 2). At SHAM, alpha and theta AP was greatest at baseline and decreased to an apparently steady state from scrotal manipulation onward. In CAST, maximum AP for theta and alpha bands also occurred at baseline, but it was followed by a transient peak during the treatment time, then by a decrease during recovery (R05, R510, R1020).

**Figure 3:**
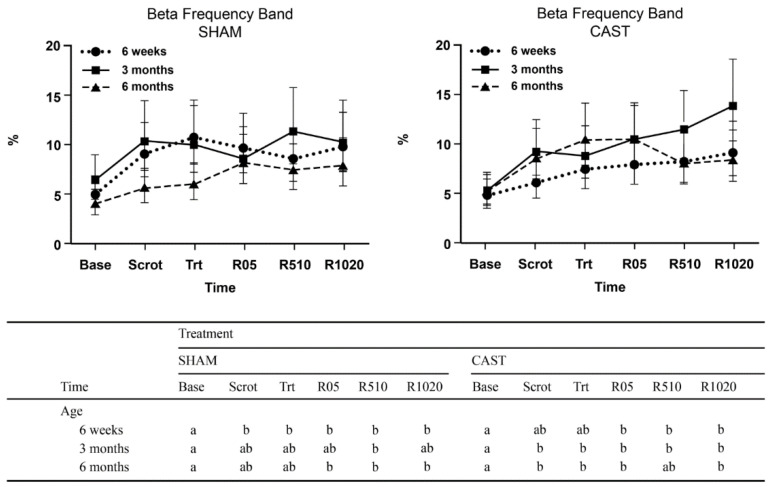
Estimated least-square means and 95% confidence intervals of relative power (%) of beta frequency bands for 6-week—(6W), 3-month—(3M) and 6-month-old—(6M) calves undergoing simulated castration (SHAM) and surgical castration (CAST) treatments. Time points evaluated consist of baseline (Base), scrotum manipulation (Scrot), treatment (Trt), immediate recovery (R05), middle recovery (R510), and late recovery (R1020). The letters (a, b) indicate a significant difference (*p* < 0.05) between time points within each combination of age category treatment.

For RP of the beta band, a three-way interaction was noted between treatment, age, and time (*p* = 0.04; Figure 3). Specifically, in 3M and 6M calves undergoing CAST, beta RP increased from baseline to scrotum manipulation time and remained high at treatment time and recovery. In contrast, when 3M and 6M calves underwent SHAM, increases in beta RP relative to baseline were only apparent for selected recovery times. For 6W calves during SHAM, beta RP increased rapidly after baseline and then plateaued for the remaining time points. At CAST, an increase in beta RP relative to baseline was also observed in 6W calves during recovery times, though of lesser magnitude.

**Figure 4:**
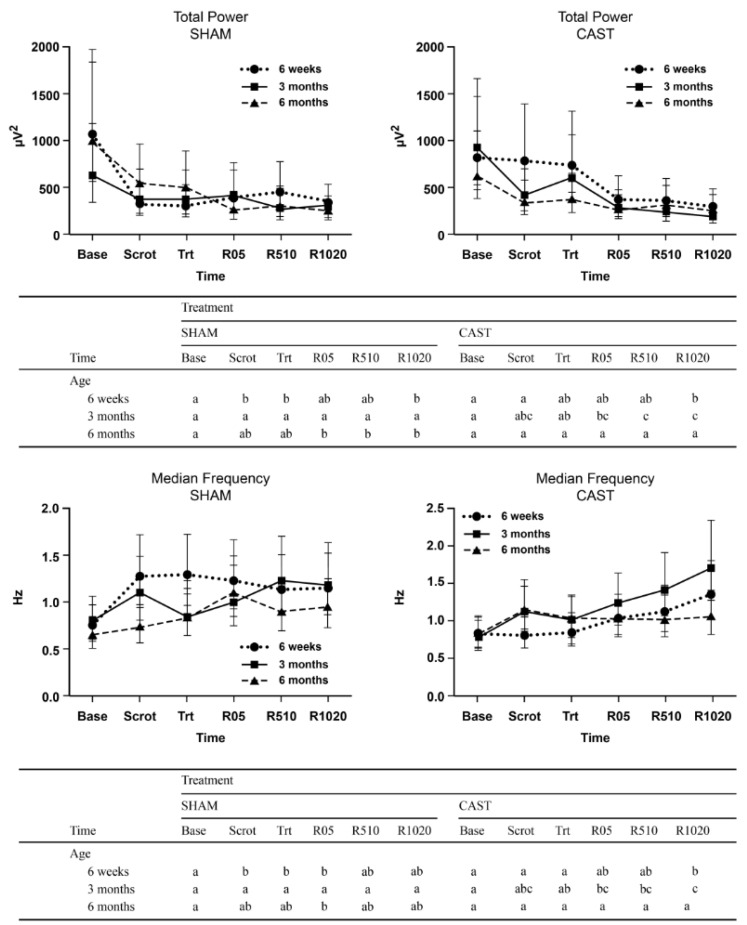
Estimated least-square means and 95% confidence intervals of total power (µV^2^) and median frequency (Hz) for 6-week—(6W), 3-month—(3M) and 6-month-old—(6M) calves undergoing simulated castration (SHAM) and surgical castration (CAST) treatments. Time points evaluated consist of baseline (Base), scrotum manipulation (Scrot), treatment (Trt), immediate recovery (R05), middle recovery (R510), and late recovery (R1020). The letters (a, b, c) indicate a significant difference (*p* < 0.05) between time points within each age category.

In the overall EEG spectrum (0.5–30.0 Hz), a three-way interaction was noted between treatment, age, and time on Ptot and on F50 (*p* = 0.03, and *p* = 0.008, respectively; Figure 4). For 6W calves undergoing CAST, Ptot decreased significantly from baseline and scrotum manipulation compared to the latest recovery time (R1020). In 3M calves undergoing CAST, there was a significant decrease in Ptot from baseline and treatment time relative to later recovery times. Interestingly, these time differences were not apparent when the 3M calves underwent SHAM. For 6M calves undergoing SHAM, a significant decrease in Ptot was observed from baseline relative to recovery. These differences were not apparent at CAST in this age group.

On F50, a three-way interaction between treatment, age, and time showed a significant increase from treatment time compared to the latest recovery time for 6W and 3M calves (but not for 6M calves) undergoing CAST. These differences were not apparent at SHAM.

### 3.2. SP Concentrations

**Figure 5:**
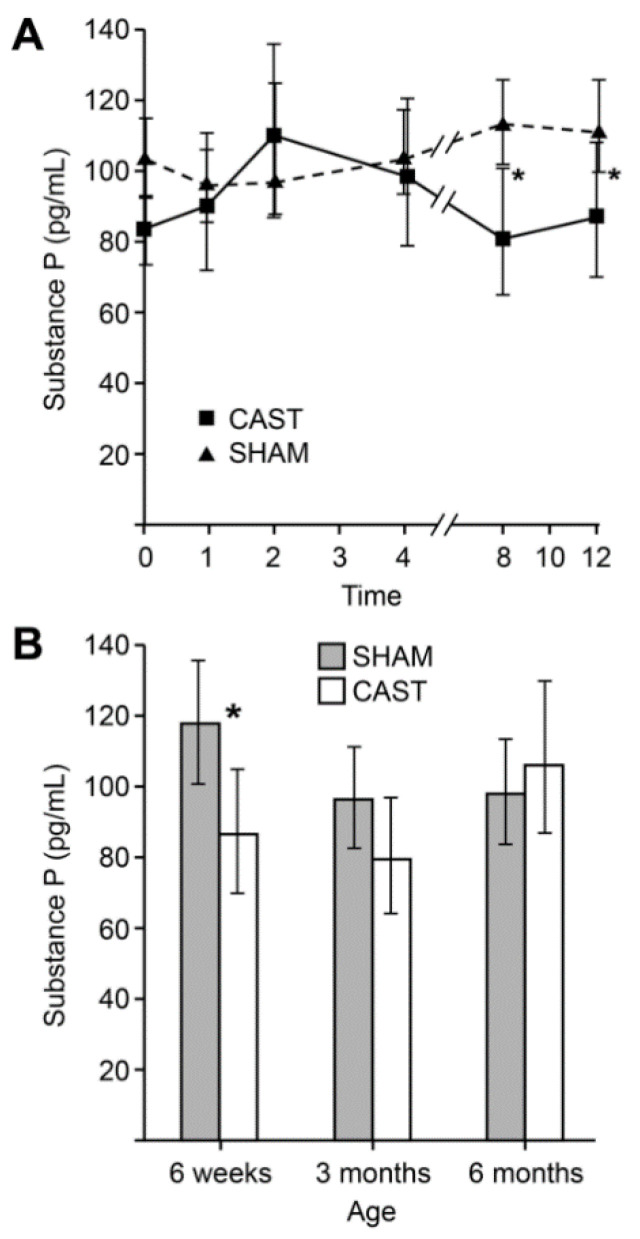
(**A**) Estimated least-square means and 95% confidence intervals of SP concentrations (pg/mL) observed at simulated castration (SHAM) and surgical castration (CAST) treatment across age groups. Time points evaluated consist of prior to treatment (0 time), and 1, 2, 4, 8 and 12 h after treatment. * Indicates a significant difference (*p* < 0.05) between the time points within each treatment. (**B**) Estimated least-square means and 95% confidence intervals of SP concentrations (pg/mL) observed at simulated castration (SHAM) and surgical castration (CAST) in each age group. * Indicates a significant difference (*p* < 0.05) between the two treatments in each age group.

Marginal evidence for two-way interactions were noted between time and treatment and between age and treatment on the concentration of SP (*p* = 0.068 and *p* = 0.066, respectively). Specifically, substance P concentrations were lower in CAST compared to SHAM at later recovery times (Figure 5A). Six-week-old calves showed lower SP concentration in CAST relative to SHAM (*p* = 0.0174; Figure 5B), though this difference was not apparent in older calves.

## 4. Discussion

The present study examined physiological responses to unmitigated castration in calves of different ages with the ultimate goal of supporting pain mitigation strategies for routine management practices like castration. The administration of local anesthesia prior to castration is legislated in several European countries [28], though it is not routine practice in cattle production systems in North America [5,6,29]. In the United States, there are several association guidelines that do include provisions for pain mitigation during castration [1,30]. The Guidelines for the Care and Use of Agricultural Animals in Research and Teaching states that “it may be desirable to inject a local anesthetic in the scrotum of older calves when surgical methods of castration are used or when the spermatic cords are crushed” [30]. In the castration guidelines set forth by the American Association of Bovine Practitioners (AABP), “veterinarians are encouraged to work with clients to advance” the use of local anesthetic during castration procedures [1]. Additionally, guidelines suggest performing castration at the youngest age practicable [1,2].

In this study, we characterized physiological responses in calves of different ages undergoing unmitigated surgical castration using EEG and SP concentrations. The EEG results of the present study are consistent with previous research investigating the acute noxiousness of routine practices using a minimal anesthesia model [14,31] and support that even calves of 6 weeks of age perceive and respond to pain. For many of the EEG variables, three-way and two-way interactions between treatment, age, and time were noted, thus indicating that the dynamics of the EEG activity during castration were for the most part age-specific and that they differed between the painful (CAST) and “painless” (SHAM) procedures. To be more precise, EEG responses on 6W calves showed an increase in beta frequency band at CAST, which seems to contradict the popular credence that younger animals are less sensitive to pain associated with castration. In EEG power spectrum analysis, a numerical increase in F50 indicates replacing the predominant high-voltage slow-frequencies (delta, theta) with low-voltage fast-frequencies (alpha, beta) during desynchronization related to nociception in animals [15]. An increase in F50 and a decrease in Ptot identified desynchronization or arousal reaction to noxious stimulation in the EEG during castration in horses anesthetized with halothane [31]. In the present study, we found a decrease of Ptot in 6W and 3M calves undergoing CAST with greater Ptot at treatment relative to late recovery. These differences were not apparent at SHAM. Since the power spectrum contains more power at lower frequencies than at higher frequencies, Ptot is preferentially sensitive to changes in such lower frequencies (e.g., delta). Therefore, a redistribution of the power to the higher frequencies (e.g., beta) as well as an increase in F50 is indicative of a shift toward faster frequencies (i.e., desynchronization).

Induced pain models in humans commonly show a consistent pattern; with more intense painful stimulation, EEG frequencies tend to increase in power and beta frequency increases relatively more than other bandwidths [32,33]. In conscious animals, noxious stimuli elicited an increase in the mid-range frequencies of the EEG [34] and EEG activity exhibited a significant shift toward faster frequencies (e.g., beta) in response to castration even in calves receiving intravenous sodium salicylate to mitigate castration-associated pain [13]. Results from the present study also support an effect of castration on increased beta frequency.

Few significant differences in EEG outcomes were noted between SHAM and CAST at the time of treatment or during early recovery. Only the younger calves (6W and 3M) showed differences in delta and beta AP at the time of treatment between the two experimental procedures. Considering that all calves underwent a simulated castration session, followed 24 h later by surgical castration, the novelty of scrotum manipulation could have been less effective in activating a strong arousal response during CAST treatment due to the partial non-painful habituation that occurred during SHAM. This can be interpreted as a reduction in perceived stimulus severity or habituation to the stimulus. Habituation occurs in all modalities of the senses and can likely result from either central or peripheral desensitization [35]. Additional factors such as distraction and stress have also been shown to influence endogenous pain modulation [36,37].

Based on EEG outcomes, our results suggest that younger calves (6W and 3M) respond differently to castration-induced pain compared to 6M calves (e.g., beta frequency band, Ptot and F50). The findings of this study are in agreement with a study conducted in calves aged < 8 weeks and > 6 months that found that older calves had the greatest magnitude of EEG desynchronization during cut-and-clamp castration, and that older calves had a greater magnitude of EEG desynchronization during cut-and-pull castration compared to younger calves [38]. The age of animals at castration has been shown to impact pain perception in other studies as well. While pain response was identified during and after castration in calves less than 1 week old [39], calves younger than two months of age castrated with knife or band castration methods in another study did not exhibit changes indicative of chronic pain in the target indicators included in the study. A study conducted in lambs under light halothane anesthesia [40] showed that the pattern of EEG responses to castration differed between 2- and 4-week-old lambs. That study indicated a qualitatively different perception of the noxious stimulus in the lambs of different ages, and the authors suggested that younger lambs perceived castration as a greater visceral noxious stimulus, whereas a greater somatic noxious stimulus component prevailed in older lambs.

The neuropeptide SP has been used to measure pain in cattle during studies on castration [22,41]. The results from the present study showed lower concentrations of SP in calves after surgical castration (CAST) relative to simulated castration (SHAM). These results contradict studies that show a significant increase in plasma SP concentration after castration [20,22], and mean SP concentration in castrated calves being significantly greater than the concentration in control calves [21]. It should be noted that the reliability of the SP assay between studies makes consistency and thus comparisons between studies challenging.

In the study by Coetzee et al. [20], SP concentrations were assessed up to 4 h after castration, while in the present study SP was measured up to 12 h after treatment. Meléndez et al. [19] observed higher SP concentration in beef calves that received oral meloxicam at 1 mg/kg P.O. compared to calves that received 0.5 mg/kg meloxicam S.C. prior to surgical castration. Vellani et al. [42] reported a significant increase in SP release 70 min after stimulation that reflected what was seen, in the present study, 1-h post-castration. These differences are not significant and can be attributed to noise and thus not repeatable. It may be that SP concentrations in plasma are “depleted” after a noxious stimulus resulting in a paradoxical decrease in circulating SP at later time points. This may also be associated with increased catabolism of SP in animals with inflammation after castration or in animals that were exposed to a painful stimulus.

Moreover, the above-mentioned studies were performed on Angus-crossbred calves that had a shorter acclimation period compared to the present study and housing consisting of dry-lot confinement followed by indoor stalls. Breed differences (beef calves compared to dairy calves) as well as different vaccination protocols might affect SP concentrations. Coetzee et al. [20] reported very high concentrations of SP compared to the results of the present study. It is possible that the abbreviated acclimation period, differences in sampling schedule, and breed difference influence SP concentrations. As a consequence, the previously mentioned conditions might have affected the further SP release associated with the castration treatment or they might be related to the less availability of SP in the bloodstream compared to the local injured area.

Among other functions, SP induces contraction of gastrointestinal smooth muscle [43]. A study on the concentration of SP in the abomasal wall reported significantly lower concentration in German Holstein cows compared to German Fleckvieh cows [44]. The overall higher plasma SP concentration observed in calves after sham castration in the present study might therefore reflect increased gastrointestinal motility that usually occurs when the animal is not experiencing a high level of distress. However, further investigations are needed.

## 5. Conclusions

In this study, EEG responses and SP concentrations were evaluated in calves of different ages undergoing unmitigated surgical castration. The EEG findings have implications for supporting the perception of pain, even in young calves, associated with surgical castration. These findings seem to contradict the notion that younger animals are less sensitive to the pain associated with castration; younger animals may just react differently to pain than older animals. In conclusion, unmitigated surgical castration resulted in a stress event even for 6-week-old calves. Further research is needed to discern whether performing castration within the first week of life would actually result in improving the welfare of the animal. Additionally, future work could explore the interactions of other management factors on pain response, such as diet and housing, as in the United States dairy system, there are some inherent differences with management related to calf age.

## Data Availability

Please contact the corresponding author with enquires.
